# Women’s Mid-Life Night Sweats and 2-Year Bone Mineral Density Changes: A Prospective, Observational Population-Based Investigation from the Canadian Multicentre Osteoporosis Study (CaM*os*)

**DOI:** 10.3390/ijerph15061079

**Published:** 2018-05-26

**Authors:** Evelyn M. M. Wong, George Tomlinson, Marsha M. Pinto, Claudie Berger, Angela M. Cheung, Jerilynn C. Prior

**Affiliations:** 1 Division of Endocrinology and Metabolism, Department of Medicine, University of British Columbia, Vancouver, BC V5Z 1M9, Canada; evelyn.mm.wong@gmail.com; 2 Division of Endocrinology and Metabolism, Department of Medicine, University Health Network and Sinai Health System, University of Toronto, Toronto, ON M5G 2C4, Canada; angela.cheung@uhn.ca; 3 Osteoporosis Program, University Health Network, Toronto, ON M5G 2C4, Canada; marshapinto@gmail.com; 4 Institute of Health Policy, Management and Evaluation, University of Toronto, Toronto, ON M5G 2C4, Canada; george.tomlinson@thebru.ca; 5 Toronto General Hospital Research Institute, Toronto, ON M5G 2C4, Canada; 6 Division of General Internal Medicine, Department of Medicine, University Health Network and Sinai Health System, University of Toronto, Toronto, ON M5G 2C4, Canada; 7 Institute of Medical Sciences, University of Toronto, Toronto, ON M5G 2C4, Canada; 8 Canadian Multicentre Osteoporosis Study Data Management Group, McGill University, Montreal, ON H4A 3S5, Canada; claudie.berger@mail.mcgill.ca; 9 Centre of Excellence in Skeletal Health Assessment, University of Toronto, Toronto, ON M5G 2C4, Canada; 10 Centre for Menstrual Cycle and Ovulation Research, University of British Columbia, Vancouver, BC V5Z 1M9, Canada; 11 School of Population and Public Health, University of British Columbia, Vancouver, BC V6T 1Z3, Canada; 12 BC Women’s Health Research Institute, Vancouver, BC V6H 2N9, Canada

**Keywords:** vasomotor symptoms, hot flashes, night sweats, menopause, women, perimenopause, osteoporosis, spinal fractures, hip fractures, bone density

## Abstract

Women’s hot flushes and night sweats, collectively called vasomotor symptoms (VMS), are maximal (79%) in late perimenopause. The evidence describing whether VMS are associated with loss of areal bone mineral density (BMD) is mixed. We examined baseline and 2-year data for 1570 randomly selected women aged 43–63 in the Canadian Multicentre Osteoporosis Study (CaM*os*), a prospective Canada-wide study; we used linear regression to assess the relationship of night sweats (VMSn) with BMD and its changes. Clinically important VMSn occurred for 12.2%. Women with VMSn were slightly younger (54.5 vs. 55.3 years, *p* = 0.02) and less likely to use sex steroid therapies (39.8% vs. 51.4%, *p* < 0.05). BMD at the lumbar spine (L1-4), femoral neck (FN) and total hip (TH) were similar between those with/without VMSn. In adjusted models, we did not find a significant association between VMSn and 2-year change in L1-4, FN and TH BMD. Age, reproductive status, weight, sex steroid therapy and smoking status were associated with 2-year change in BMD. Incident fractures over 2 years also did not differ by VMSn. Our analyses were restricted to VMSn and may not truly capture the relationship between VMS and BMD. Additional research involving VMS, bone loss and fracture incidence is needed.

## 1. Introduction

Vasomotor symptoms (VMS), encompassing both daytime hot flushes/flashes and night sweats during sleep (VMSn), are experienced by some midlife women with regular menstrual cycles [1] and peak in prevalence at approximately 79% in late perimenopause [2]. The first research suggesting a relationship between bone health and VMS was a retrospective clinical case-control study in menopausal women with osteoporosis versus age-matched controls; those with vertebral fractures were 35% more likely to recall VMS and describe them as problematic and persistent [3]. Likewise menopausal women from the Women’s Health Initiative study who had never used ovarian hormone therapy (OHT) experienced an almost two-fold increased incidence of hip fracture in those with the most frequent and intense VMS at baseline [4]. Thus there is evidence suggesting that clinically problematic VMS are related to an increased incidence of fracture.

However, the bone turnover, hormonal and areal bone mineral density (BMD) mechanisms through which VMS may relate to fracture risk are unknown. A Swedish population-based cross-sectional study (Eindhoven Perimenopausal Osteoporosis Study) in over 5000 perimenopausal women showed lower baseline (1994-5) L1-4 BMD values related to the frequency of VMS; this relationship showed a significant dose-response (*p* < 0.0001) [5]. Also a small study of women in their mid-30 s with infertility and VMS but primarily regular cycles found that higher bone resorption marker levels were more strongly related to night sweats (VMSn) than to daytime VMS [6]. Although four prospective studies have evaluated an association between BMD change and VMS experiences [7,8,9,10], only one showed a significant inverse relationship. However, some of these results were confounded by various therapies (Appendix A, Table A1).

Since the rate of BMD loss in late perimenopause exceeds that in the first years of menopause [11], it is important to study whole populations to learn whether more intense VMS might add to that lifecycle-related BMD loss. Thus the objectives of this study in women aged 40–60 years old at baseline in the Canadian Multicentre Osteoporosis Study (CaM*os*), a population-based prospective cohort study [12], were to: (1) to evaluate clinically important (moderate to severe in frequency and intensity) versus absent-minimal/mild VMSn at baseline related to 2-year changes in BMD at lumbar spine (L1-4), femoral neck (FN) and total hip (TH) sites; and; (2) to assess VMSn categories related to the rate of 2-year incident fractures.

## 2. Materials and Methods

This study used a prospective cohort design to examine baseline VMSn and bone health (two-year change in bone and incident fracture) in a random sample of the population.

### 2.1. Participants

CaM*os* is a prospective study of skeletal health in a randomly selected non-institutionalized population of men and women ≥25 years drawn from within a 50 km radius of nine Canadian cities (Vancouver, Calgary, Saskatoon, Toronto, Hamilton, Kingston, Quebec City, St. John’s, and Halifax) and initiated in 1995–1997. A detailed description of the purpose, methodology and sampling framework for CaM*os* is available elsewhere [12]. Ethics approval was obtained through the Review Boards of each participating centre and at the coordinating centre, McGill University, Montreal, Quebec, Canada.

We examined 1570 women, aged 43–63 at this study’s baseline (1998–2000) with 2-year follow-up (1998–2000 to 2000–2002). A total of 108 women were excluded in the analysis if they did not have paired data on the L1-4, TH or FN measurements or had received more than three months of the following bone-modifying medications over the two-year period: alendronate, calcitonin, clodronate, etidronate, fluoride, raloxifene, risedronate or tamoxifen.

### 2.2. Measures

Data collection consisted of an in-person interviewer-administered questionnaire and a number of physical measurements (see below). Data collected in an interviewer-administered questionnaire included sociodemographic, lifestyle, reproductive, medical history, nutritional, and lifestyle information. Questions about VMS during sleep (VMSn) were added to the CaM*os* questionnaire at Year 3 (baseline for this cohort). Daytime VMS were not included because VMSn sufficient to cause wakening were perceived to cause greater physiological disruption [6,13]; daytime VMS were also not added to limit respondent burden. Self-reported data on VMSn frequency and intensity over the last two weeks were collected at baseline and after 2-years. Women reporting VMSn at baseline of at least moderate intensity for ≥3 times in the last 2 weeks were grouped as having clinically important VMSn; those without VMSn or with mild/less frequent night sweat experiences formed the control group.

Reproductive status was classified as follows: *premenopause* if women reported menses in the past year or age <52 years and underwent a hysterectomy without oophorectomy or with unilateral oophorectomy; *natural menopause* if women reported ≥1 years without menses, or if age ≥52 years and underwent a hysterectomy without oophorectomy or with unilateral oophorectomy, or if women reported ≥1 year without menses before hysterectomy/oophorectomy; and, *surgical menopause* if bilateral oophorectomy [14,15]. Using these definitions, women in the *menopausal transition/perimenopause* were included in the premenopause category. Women’s self-reported information on participation in a regular activity or program either on their own or in a formal class was used to assess physical activity. Smoking was classified into current, past and never smokers because both past and current smoking are associated with BMD and increased fracture risk in cross-sectional and longitudinal studies, although that risk is lower in past smokers [16,17]. Current cigarette use is also associated with an increased risk for clinically important VMS [18]. Because ovarian hormone therapy or combined hormonal contraceptives are related to BMD, this variable was used in the linear models. Weight was included as a covariate as it could positively confound our results because higher body mass index or obesity, perhaps due to insulation or wider estrogen fluctuations, has been associated with more frequent and intense VMS [19] and higher fracture risk (hazards ratio 1.16 [1.09,1.23]) [20].

Physical measurements included height, weight, and BMD by dual X-ray absorptiometry (DXA). Areal BMD measurements were obtained at the spine in levels L1-4, FN and TH at baseline and after 2-years. A European spine phantom was measured systematically at least once per BMD measurement-year at each centre; this allowed researchers to assess the linearity of data from all the centres and adjust all data to a common reference [21]. Since some of the BMD instruments were manufactured by Lunar (General Electric Company, Boston, MA, USA) and some by Hologic (Marlborough, Massachusetts, USA), all data were converted to Hologic equivalent values [22,23]. Change in BMD was expressed as 2-year percentage change, calculated as 100 × (2-year minus baseline)/baseline.

Every 12 months after the baseline assessment, each participant received a short follow-up postal questionnaire asking for reports of fragility fractures (fractures occurring with the same or less force than a fall from a standing height) at all sites excluding the skull, hands and feet. If a participant reported a fracture, the site study coordinator obtained corroborating evidence to medically/radiographically confirm the presence of a fragility fracture [24].

### 2.3. Statistical Analysis

Frequency distribution and measures of central tendency and dispersion were calculated to describe population characteristics. Means and standard deviations or medians and interquartile ranges were reported, as appropriate; counts and percentages were reported for categorical variables. Tests of equal means at baseline in those with and without clinically important VMSn used *t*-tests and tests of equal frequency distributions used chi-squared tests, with one exception: the presence of fragility fractures was compared with Fisher’s exact test.

Multivariable linear regression analyses were used to evaluate associations between 2-year percent change in BMD at each site between women with clinically important VMSn versus those without. Models were adjusted for age or reproductive status (in separate models as they are correlated), weight, physical activity, estrogen-based therapy use, smoking status and family history of fracture. Age and weight were modelled with a 3-degree of freedom natural cubic spline to allow a nonlinear relationship to BMD changes. Two additional models were checked for an interaction between estrogen therapy use and VMSn because estrogen therapy is commonly used to treat menopausal VMS [25] and between reproductive status and VMSn to deal with the variable prevalence of VMSn across the reproductive changes from very early perimenopause into the early years of menopause [15]. Cox proportional-hazards regression was used to assess the association of VMSn category with incident fracture. We note that the linear regression analyses entailed examining both adjusted and unadjusted effects of VMSn at three separate bone sites, with a primary model (with either age or reproductive status) and two adjusted models with interactions, making a total of 15 regression models; we report all results for effects of VMSn on BMD without adjusting p-values. All analyses were performed using R Statistical Software (R3.4.4, 2018, R Foundation for Statistical Computing, Vienna, Austria).

## 3. Results

### 3.1. Population Characteristics

The prevalence of clinically important VMSn was 12.2% (n = 191). Table 1 describes the study population by their experience of clinically important VMSn or not. The mean (±SD) age of menopause in the population was 46.1 ± 7.3 years.

Women with important VMSn were younger (54.5 ± 4.6 vs 55.3 ± 5.2 years, *p* = 0.020) and less likely to take estrogen-based sex steroid therapies (39.8% vs. 51.4%, *p* = 0.003). A greater proportion of current smokers were seen in the group with important VMSn (18.8% vs. 12.6%, *p* = 0.048). No other statistically significant differences were seen in the baseline characteristics of the women in this midlife population.

### 3.2. Cross-Sectional Bone Mineral Density Values at Baseline and Two Years

Cross-sectional baseline BMD values at L1-4, FN and TH sites were similar between those with clinically important VMSn and without. The same was true of all BMD values after two years (Table 1).

### 3.3. Two-Year Bone Mineral Density Changes

**Figure 1:**
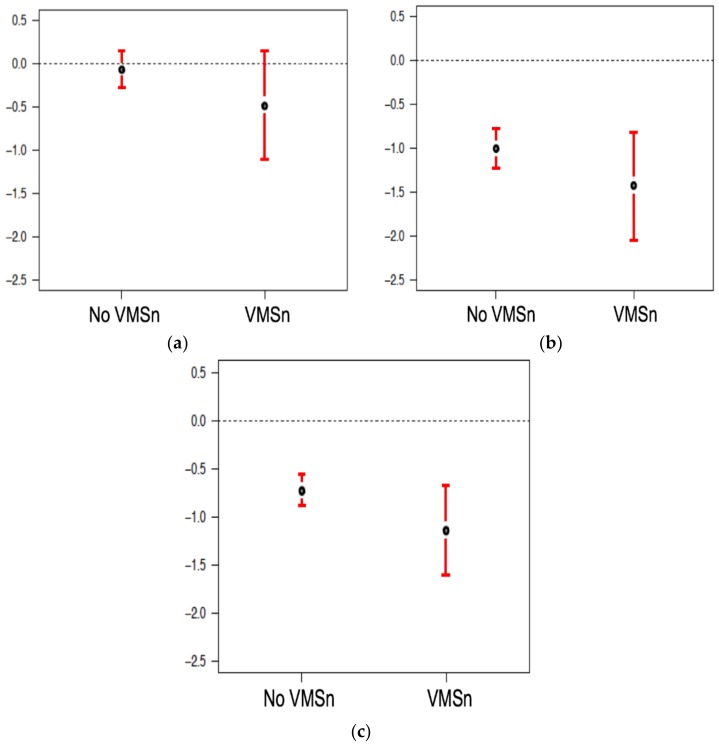
Two-year mean Percentage Changes in areal Bone Mineral Density values (BMD 95% CI) by Experience of Clinically Important Night Sweats (VMSn) or mild or absent VMSn (No VMSn) in Women ages 43–63 in the Canadian Multicentre Osteoporosis Study (CaM*os*) at: (**a**) L1-4 spine BMD; (**b**) FN BMD; (**c**) TH BMD sites.

In unadjusted comparisons, women with clinically important VMSn experienced a non-significantly greater two-year L1-4 BMD loss (mean difference −0.42%, 95% CI (−1.08, 0.24), *p* = 0.211) (Figure 1a). In simple, unadjusted comparisons, clinically important VMSn were also not associated with 2-year BMD changes at the FN and TH (mean differences: −0.42%, 95% CI (−1.08, 0.24), *p* = 0.208; and −0.41%, 95% CI (−0.90, 0.08), *p* = 0.099, respectively) (Figure 1b,c).

In adjusted models of 2-year L1-4 BMD change by the presence of clinically important VMSn or not, there was no relationship (Table 2). Age and reproductive status (in separate models), weight, sex steroid therapy use, and smoking status accounted for variations in L1-4 BMD. Decreases in L1-4 BMD were seen up to and around the average natural menopausal age of 52 years (from 3-degree association of age with L1-4). Model-predicted percent changes in L1-4 BMD were around −0.5% (loss) for weights up to 75 kg, then increased to +1% (gain) for a weight around 110 kg. Similarly, in multivariable linear regression models of BMD change (Table 3 and Table 4), the presence of clinically important VMSn was not associated with different 2-year changes in BMD values at the FN or TH.

Age, but not weight, was associated with change in FN and TH BMD in a similar pattern as at L1-4. Those with VMSn using sex steroid therapy had greater changes in TH BMD outcomes than those not using sex steroid therapy, with changes that were 1.23% higher (95% CI (0.23–2.23), *p* = 0.013), an interaction not seen with FN (*p* = 0.74) or L1-4 (*p* = 0.81) BMD change. Across all regression models fitted, the largest R^2^ was 5%, so changes in BMD over the two years are largely not explained by the variables in these models.

### 3.4. Two-Year Incidence of Fragility Fractures

In women with clinically important VMSn, 4 (2.1%) experienced an incident fracture while 36 (2.6%) of those with absent or mild VMSn experiences suffered an incident fracture (*p* = 1.0). VMSn was not found to be associated with the incidence of fragility fracture at any site {hazard ratio (HR) = 0.96, 95% CI (0.34, 2.74), *p* = 0.94}.

## 4. Discussion

To date, our study is the first population-based study to examine prospective BMD change by the experience of clinically important vasomotor symptoms occurring during sleep (night sweats, VMSn). We did not find a significant association between clinically important VMSn and 2-year BMD change at any of the L1-4, TH or FN sites that were not already accounted for by other known modifying factors, although there may have been small non-significant trends towards greater BMD loss with VMSn at all three sites. Our results are consistent with several studies [8,9,10]. Specifically, increased weight and use of estrogen-based sex steroid therapy are associated with positive L1-4 BMD changes. Losses in L1-4 BMD were seen in women up to and around the average natural menopausal age of 52 years and increases were seen in older women. This effect may be because the greatest losses in L1-4 BMD occur in the last perimenopausal and the first menopausal years [11]; lumbar spine (L1-4) BMD losses typically plateau or decrease thereafter [26,27]. In addition, women who were previous smokers experienced a significant increase in L1-4 BMD, which is not unexpected as previous studies have shown that active smoking is associated with lower BMD and smoking cessation is associated with improvements in BMD [28,29].

In the models evaluating 2-year percent change in FN and TH BMD, there was no significant independent effect of clinically important VMSn. Age accounted for variations in BMD at both sites, similar to the L1-4 site. When examining 2-year percent change in TH BMD, there was no significant independent effect of either clinically important VMSn or estrogen-based steroid therapy, but the interaction term was significant. This interaction however was not seen at other BMD sites and may more likely represent a chance finding.

Our results are generally consistent with Salamone et al. [8] who did not find significant differences in annualized rates of change in BMD at the L1-4 or TH when comparing women with or without hot flushes respectively, and Tuomikoski et al. [10] who examined 114 women in an observational study where annualized rates of lumbar spine and bilateral TH BMD change were not different between women with no, mild, moderate or severe hot flushes (Appendix A, Table A1). On the other hand, a study by Naessen et al. [7] assessing 40 peri- and menopausal women (aged 45–56) on ovarian hormone therapy vs. untreated, age-matched controls did find a difference in BMD change at the forearm in women with frequent daily sweating (Appendix A, Table A1).

Our cross-sectional analysis comparing L1-4, TH, and FN BMD between groups at baseline and at 2-year follow-up were consistent with the Rancho Bernardo study [30]. However, this latter population was different from our population as women were much older (mean age 73 years, range 47–97) with a higher frequency of night sweats (36.1% vs. 12% in our population), and additionally the data were not adjusted for hormone therapy [30]. On the other hand, multiple cross-sectional studies have examined the relationship between BMD and VMS and found lower BMD with any or more severe VMS [3,5,8,30,31,32]. Variations in study methodology, such as subcategorization of reproductive status into early or late peri- or menopause, VMS recall over various time periods (e.g., 30 days in Ozkaya et al. [33]), and the inclusion of both hot flushes and night sweats may have contributed to differences.

We also did not find a difference in the incidence of fracture between groups in our study; however, this was not unexpected given the short time frame (2 years) and relatively young population. Crandall et al., in a large cohort, did find an increased rate of hip fractures in menopausal women with moderate-severe VMS over an average of 8.2 years of follow up (hazard ratio 1.78, 95% CI (1.20–2.64)), in addition to lower cross-sectional FN and LS BMD [4].

This is the first and only population-based longitudinal study to date examining the association between night sweats and the rate of bone loss. The CaM*os* participants were selected randomly from the Canadian population and represent an age-, sex- and region-specific sample. The strength of our study is that it is a large population-based longitudinal study examining prospective BMD change in midlife women by VMSn experiences in the last two weeks. We did not find evidence to support the exclusive use of night sweats severity and frequency to predict BMD loss; however the combination of daytime and nighttime symptoms may change these results.

There are several reasons that could account for differences between our study and others. Our results are restricted to a short time frame (the 2 weeks prior to the questionnaire) and to night sweats alone (VMSn); thus we may not have truly captured the relationship between daytime VMS and BMD. Furthermore, given the lack of a gold standard to define the intensity and frequency VMS, variations in VMS measurement may have contributed to differences in results. We defined reproductive status based on presumed hormonal changes in the midlife period and therefore may have misclassified participants. Also, Naessen et al. found differences in change in forearm BMD, a site of significant BMD loss in menopause [7], but we did not examine this site in our study.

## 5. Conclusions

Our population-based longitudinal study did not find a significant relationship between clinically important peri- and menopause-related night sweats (VMSn) and BMD loss or fracture incidence. Further longitudinal studies of longer duration that include daytime hot flushes/flashes as well as VMSn may better elucidate the true relationship between VMS and fragility fracture incidence, the clinically relevant outcome. In addition, markers of bone quality such as trabecular bone score or high resolution peripheral quantitative computed tomography would be helpful to better understand the potential relationship between VMS, bone changes and fractures.

## Figures and Tables

**Figure 2:**
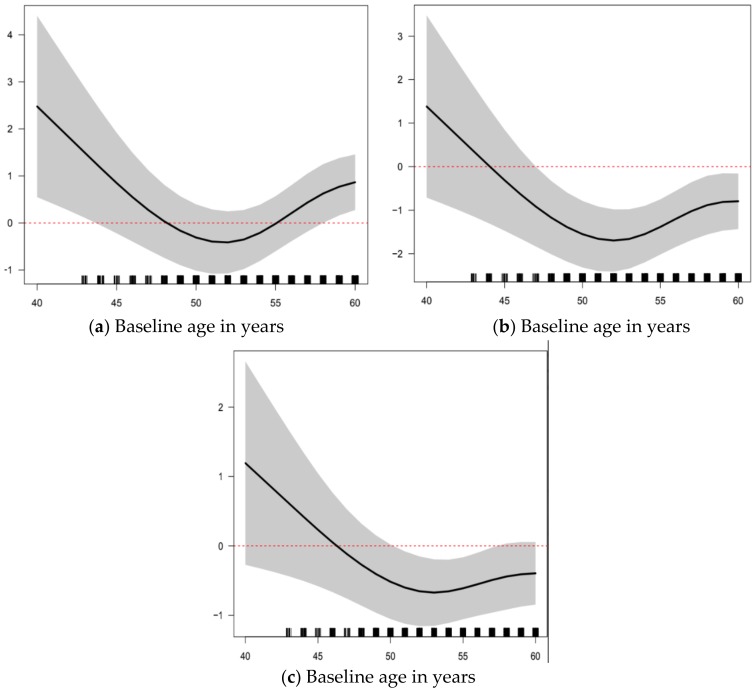
Two-year Percentage Change in areal Bone Mineral Density in Relationship to Baseline Age; Percent change in BMD (95% CI): (**a**) Lumbar Spine (L1-4); (**b**) Femoral Neck; and (**c**) Total Hip in Women ages 43–63 in the Canadian Multicentre Osteoporosis Study (CaM*os*) (n = 1570).

**Figure 3:**
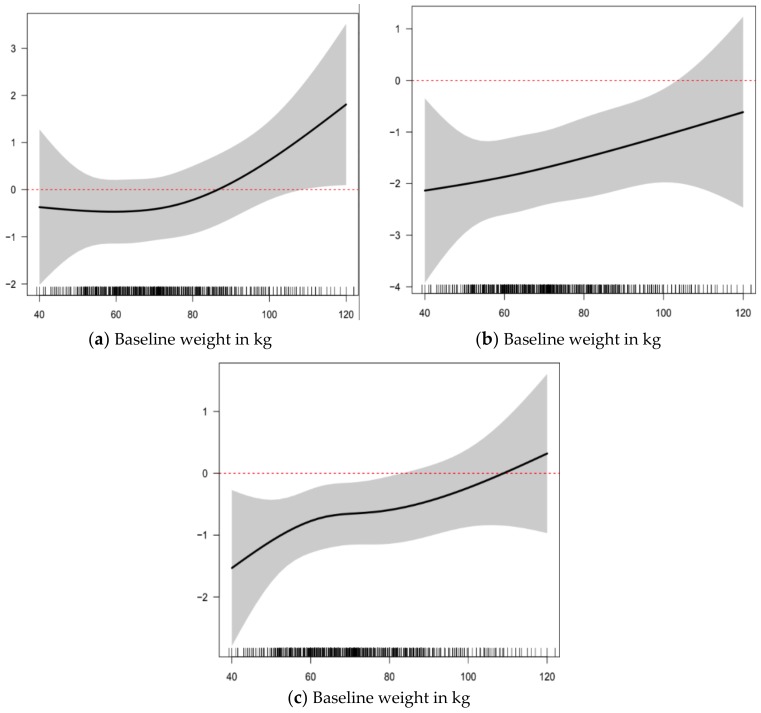
Two-year Percentage Changes in areal Bone Mineral Density (BMD 95% CI) in Relationship to Baseline Weight; Percent change in: (**a**) Lumbar Spine (L1-4); (**b**) Femoral Neck; and (**c**) Total Hip in Women ages 43–63 in the Canadian Multicentre Osteoporosis Study (CaM*os*) (n = 1570).

**Table 1:** Population Characteristics at Baseline of Women ages 43-63 in the Canadian Multicentre Osteoporosis Study.

Characteristic	Absent/Mild VMSn	Clinically Important VMSn	* *p*-Value
N (%)	1379 (87.8)	191 (12.2)	
Age (years), mean (SD)	55.3 (5.2)	54.5 (4.6)	0.020
Weight (kg), mean (SD)	71.2 (14.1)	72.1 (15.6)	0.457
Height (cm), mean (SD)	161.0 (6.1)	160.9 (6.3)	0.833
Body mass index kg/m^2^, mean (SD)	27.5 (5.2)	27.8 (5.7)	0.380
Reproductive status, n (%)			0.261
Premenopause	358 (26.0)	51 (26.7)	
Natural menopause	866 (62.8)	126 (66.0)	
Surgical menopause	155 (11.2)	14 (7.3)	
Mean age at menopause years, mean (SD)	46.1 (7.2)	46.3 (7.9)	0.773
Sex steroid therapy, n (%)	708 (51.4)	76 (39.8)	0.003
Regular physical activity, n (%)	706 (51.2)	85 (44.5)	0.098
Smoking status, n (%)			
Current	174 (12.6)	36 (18.8)	0.047
Past	512 (37.2)	71 (37.2)	
Family history of fracture, n (%)	621 (45.0)	77 (40.3)	0.249
Family history of osteoporosis, n (%)	243 (17.6)	40 (20.9)	0.308
Presence of Fragility fracture, n (%)	36 (2.6)	4 (2.1)	0.810
Baseline BMD (g/cm^2^), mean (SD *)			
L1-4	0.996 (0.151)	0.991 (0.147)	0.676
FN	0.762 (0.111)	0.763 (0.117)	0.909
TH	0.918 (0.127)	0.921 (0.129)	0.797
BMD (g/cm^2^) at Year 2 mean (SD *)			
L1-4	0.995 (0.152)	0.986 (0.150)	0.452
FN	0.754 (0.111)	0.752 (0.114)	0.771
TH	0.911 (0.128)	0.911 (0.130)	0.976

* *p*-values comparing groups come from *t*-tests for continuous variables and chi-squared tests for categorical variables, with one exception: the presence of fragility fractures was compared with Fisher’s exact test.

**Table 2:** Multivariable Linear Regression Models without interactions for 2-year Percentage Change in areal BMD (95% confidence intervals) at **Lumbar Spine** (L1-4) in Women ages 43–63 in the Canadian Multicentre Osteoporosis Study (CaM*os*).

Baseline Variables	2-Year Percent Change in BMD (95% CI)	
In Model	Model 1	Model 2
**Clinically important VMSn (vs. No or Mild VMSn)**	−0.138 (−0.770, 0.493), *p* = 0.669	−0.309 (−0.942, 0.324), *p* = 0.339
Age (years)	(Figure 2a)	NA
Weight (kg)	(Figure 3a)	(Figure 3a)
Reproductive Status (vs. Premenopausal)		
Naturally Menopausal	NA	1.117 (0.612, 1.622), *p* < 0.01
Surgically Menopausal	NA	0.933 (0.128, 1.738), *p* = 0.024
Regular physical activity (vs. None)	−0.352 (−0.776, 0.072), *p* = 0.105	−0.320 (−0.746, 0.105), *p* = 0.141
Family history of fracture (vs. None)	0.030 (−0.391, 0.450), *p* = 0.891	0.039 (0.384, 0.463), *p* = 0.856
Sex steroid therapy (vs. Never)	0.491 (0.048, 0.933), *p* = 0.030	0.330 (−0.121, 0.780), *p* = 0.152
Smoking status (vs. Never)		
Current	0.106 (−0.545, 0.756), *p* = 0.751	−0.031 (−0.685, 0.623), *p* = 0.928
Past	0.567 (0.109, 1.024), *p* = 0.016	0.553 (0.094, 1.011), *p* = 0.019
Overall R^2^	0.046	0.035
Adjusted R^2^	0.038	0.027
Residual standard error	3.959 (df = 1376)	3.982 (df = 1377)
F statistic	5.552 * (df = 12; 1376)	4.493 (df = 11; 1377)

* *p* < 0.01. Analyses were also adjusted by including cubic splines for age and weight. There are no readily interpretable estimates or *p*-values for these variables, so they are omitted from the tables.

**Table 3:** Multivariable Linear Regression Models without interactions for 2-year Percentage Change in areal BMD (95% confidence intervals) at **Femoral Neck** in Women ages 43–63 in the Canadian Multicentre Osteoporosis Study (CaM*os*).

Baseline Variables	2-Year Percent Change in BMD (95% CI)	
In Model	Model 1	Model 2
**Clinically Important VMSn**	−0.208 (−0.889, 0.473), *p* = 0.550	−0.289 (−0.970, 0.391), *p* = 0.406
Age (years)	(Figure 2b)	NA
Weight (kg)	(Figure 3b)	(Figure 3b)
Reproductive Status		
Naturally Menopausal	NA	−0.012 (−0.554, 0.531), *p* = 0.967
Surgically Menopausal	NA	0.657 (−0.207, 1.521), *p* = 0.137
Regular activity	0.053 (−0.404, 0.510), *p* = 0.822	0.069 (−0.388, 0.527), *p* = 0.767
Family history of fracture	0.091 (−0.381, 0.606), *p* = 0.655	0.117 (−0.376, 0.609), *p* = 0.643
Sex steroid therapy	0.146 (−0.331, 0.623), *p* = 0.550	0.004 (−0.480, 0.488), *p* = 0.988
Smoking status		
Current	−0.371 (−1.070, 0.329), *p* = 0.300	−0.405 (−1.107, 0.297), *p* = 0.259
Past	0.113 (−0.381, 0.606), *p* = 0.655	0.117 (−0.376, 0.609), *p* = 0.643
Overall R^2^	0.016	0.009
Adjusted R^2^	0.008	0.001
Residual standard error	4.273 (df = 1379)	4.270 (df = 1369)
F statistic	1.906 ** (df = 12; 1368)	1.173 (df = 11; 1369)

** *p* < 0.05.

**Table 4:** Multivariable Linear Regression Models without interactions for 2-year Percentage Change in areal BMD (95% confidence intervals) at **Total Hip** in Women ages 43–63 in the Canadian Multicentre Osteoporosis Study (CaM*os*).

Baseline Variables	2-year Percent Change in BMD (95% CI)	
In Model	Model 1	Model 2
**Clinically important VMSn**	−0.393 (−0.873, 0.088), *p* = 0.110	−0.440 (−0.920, 0.041), *p* = 0.074
Age (years)	(Figure 2c)	NA
Weight (kg)	(Figure 3c)	(Figure 3c)
Reproductive Status		
Naturally Menopausal	NA−	−0.188 (−0.569, 0.193), *p* = 0.334
Surgically Menopausal	NA−	−0.337 (−0.942, 0.267), *p* = 0.275
Regular activity	−0.137 (−0.459, 0.185), *p* = 0.405	−0.151 (−0.472, 0.171), *p* = 0.359
Family history of fracture	0.009 (−0.310, 0.328), *p* = 0.956	0.009 (−0.311, 0.329), *p* = 0.958
Sex steroid therapy	0.322 (−0.014, 0.657), *p* = 0.061	0.286 (−0.054, 0.625), *p* = 0.100
Smoking status		
Current	−0.197 (−0.690, 0.296), *p* = 0.435	−0.189 (−0.682, 0.305), *p* = 0.455
Past	0.052 (−0.295, 0.398), *p* = 0.770	0.060 (−0.286, 0.405), *p* = 0.736
Overall R^2^	0.016	0.011
Adjusted R^2^	0.007	0.003
Residual standard error	2.965 (df = 1342)	2.971 (df = 1343)
F statistic	1.809 ** (df = 12; 1342)	1.416 (df = 11; 1343)

** *p* < 0.05.

## Abbreviations

The following abbreviations are used in this manuscript:

BMD	areal Bone Mineral Density
CaM*os*	Canadian Multicentre Osteoporosis Study
FN	Femoral neck
HR	Hazards ratio
Kg	kilograms
L1-4 or, if unknown segments, LS	Lumbar spine
OHT	ovarian hormone therapy
TH	Total hip
VMS	Vasomotor symptoms = hot flushes/flashes and night sweats
VMSn	Vasomotor symptoms occurring during sleep, Night sweats

## Appendix A

**Table A1:** Prospective Studies of Bone Mineral Density Change in Perimenopausal and Menopausal Women Related to Experiences of Vasomotor Symptoms that were moderate-severe or interfered with their lives.

First Author/Year	Number	Characteristics	BMD Site	Duration	Rate of Change in BMD (VMS vs. Controls)
Naessen, 1992 [7]	40	Swedish general population sample Age 51.3 ± 0.3 (SEM) year Sweating episodes/day by questionnaire	Distal radius	24 mo	~9% vs. 4%/year mean difference 4.3%/year, 95% CI (0.7–7.8), *p* = 0.023
Salamone, 1998 [8]	290	USA—Women’s Healthy Lifestyle Project 47.2 ± 2.0 years Y/N to Interfering Hot flushes (yes in 14%) 40% had only night sweats—questionnaire	Lumbar Spine Total Hip	30 mo.	−0.33 ± 1.1%/year vs. −0.26 ± 1.1%/year, *p* = 0.698 0.13 ± 0.9%/year vs. −0.11 ± 1.1%/year, *p* = 0.15
Huang, 2007 [9]	2418	USA Raloxifene trial Analyzed treatment and placebo together VMS no/mild vs., mod./severe (n = 36) by questionnaire	Lumbar Spine	36 mo.	−0.17%/year (−1.02, 0.68) vs. 0.36%/year (0.23, 0.48) *p* = 0.84
Tuomikoski, 2014 [10]	114	Finland Menopausal, 71.3 ± 0.3 years VMS weekly score (per Sloan)	Lumbar Spine Left Total Hip Right Total Hip	75 mo.	−0.3 ± 0.3% vs. −0.4 ± 0.3%/year −0.6 ± 0.2% vs. −0.7 ± 0.3%/year −0.8 ± 0.2% vs. −0.8 ± 0.2%/year (NS for all)
